# Tuberculosis (T.B.) masquerading as tumor. An 8-year study on 25 cases of long bone tuberculosis presenting as tumors

**DOI:** 10.1051/sicotj/2020011

**Published:** 2020-05-28

**Authors:** Yash Wagh, Rajeev Reddy, Manit Gundavda, Manish Agarwal, Vikas M Agashe, Supreet Bajwa

**Affiliations:** 1 Department of Orthopedics, P D Hinduja Hospital and Medical Research Centre Mahim, Mumbai 400016 India; 2 Dr Agashe’s Maternity and Surgical Nursing Home Kurla, Mumbai 400070 India

**Keywords:** Tuberculosis, Long bone, Tumour, Biopsy

## Abstract

*Aim*: To highlight radiological features and emphasize the need for tissue diagnoses to confirm bone pathology. Tuberculosis is known to present without constitutional symptoms and with unconventional imaging features mimicking sarcomas as shown in our series of 25 patients; where the imaging and biopsy protocols at our institute helped to solve these diagnostic dilemmas. *Material and methods*: We retrospectively analyzed clinical and radiological features and tissue diagnoses in 25 patients referred to the department of orthopedic oncology with radiological suspicion of tumor. *Results*: Only 7 patients had cultures positive for *Mycobacterium Tuberculosis* T.B. Radiological features suggestive of infection were Metaphyseal and joint involvement, permeative lesions, absence of Codman’s triangle, and soft tissue mass suggestive of a cold abscess. The predictive accuracy of the orthopedic oncologist was 60% and musculoskeletal radiologist was 72% (based on radiology). Final diagnosis is 100% confirmed on histopathology. *Conclusion*: Diagnosis based primarily upon imaging is a wrong approach. A multimodal approach to differentiating tuberculous bone infections from sarcomas is essential.

## Introduction

Tuberculosis (T.B.) affects on an average 2.79 million people in India annually, of this about only 10–15% affect the osteoarticular regions of the body [1]. Majority of these infections affect the spine and smaller bones of the body [2], where the clinical and radiological picture of commonly affected osteoarticular regions is well defined. They, however, pose a great clinical and radiological dilemma when they affect the long bones of the body, a relatively uncommon site of presentation. Lack of constitutional signs and symptoms of tuberculosis and non-characteristic radiological features add to the diagnostic dilemma [3].

Although a large amount of literature describes clinical and radiological features of sarcomas and tuberculous osteomyelitis, none directly mentions any features to directly compare and differentiate long bone tuberculous osteomyelitis from bone sarcomas.

Tuberculous infection of the long bones can mimic benign tumors or locally aggressive tumors like giant cell tumors and at times even malignant tumors like osteogenic sarcoma or chondrosarcomas. Tulli mention that tuberculous osteomyelitis is so rare in long bones that it fails to attract the attention of the clinician [4].

It is this presentation which makes it difficult to distinguish these lesions from a sarcoma [5].

With very sparse knowledge and literature on the clinical and radiological aspects of tuberculosis affecting long bones, there is a need to bridge the gap, which will enable the correct diagnosis and management of these patients, in a timely fashion.

The aim of this study was to highlight the radiological features of osteoarticular tuberculosis and emphasize the need for tissue diagnoses (for cultures as well as histopathology) to differentiate these lesions from sarcomas. Tuberculosis is known to present without constitutional symptoms and with unconventional imaging features, thus mimicking sarcomas.

## Materials and methods

We retrospectively analyzed the clinical and radiological features along with tissue diagnoses in 25 patients. These patients were referred to the division of orthopedic oncology at a tertiary referral hospital in Mumbai, India, upon the identification of a bone lesion. All 25 patients were evaluated clinically and advised radiological imaging based on primary suspicion of a bone tumor. After evaluating the images, all patients underwent a planned biopsy for tissue diagnosis. As part of our protocol, all samples were evaluated for histopathology as well as microbiology cultures. Those patients who underwent imaging and tissue diagnoses at an outside hospital were advised to submit the same at our hospital. These images and histopathology slides were then reviewed by our team of musculoskeletal radiologists and pathologists.

The demographic data, clinical presentation, radiological imaging, location of the lesion, and histopathological tissue diagnosis reports were entered into a spreadsheet for analysis.

A protocol was formulated by our institute which aims to reduce these diagnostic dilemmas based upon extensive histopathological and radiological features differentiating tuberculosis from tumor.

## Results

Twenty five patients that were referred to our institute with suspicion of bone tumor were evaluated by the orthopedic oncologist in the outpatient department.

Based on initial screening, 15 out of the 25 patients were suspected to have bone infection as a differential diagnosis. Further review of radiological features by the musculoskeletal radiologist suspected possibility of infection in 18 out of the 25 patients.

Of the 18 patients with suspected infection, microbiological studies confirmed infection in 5 patients with positive Mycobacterium Growth Indicator Tube (MGIT) for *Mycobacterium tuberculosis.* Two patients had a culture positive for tuberculosis at the end of 6 weeks. None of the patients demonstrated bacilli on Acid fast bacilli staining at out institute.

However, all 25 patients had positive histopathological diagnosis confirming epithelioid granuloma, caseous necrosis giant cells, and predominantly lymphocytes.

Four patients had imaging done from other centers, and were provisionally reported as tumors, were reviewed by musculoskeletal radiologists at our center. As a protocol at our institute, all the patients with osteoarticular lesions undergo biopsy to confirm tissue diagnosis. Only 2 of the 25 patients had undergone a biopsy prior to referral.

A diagnosis of osteoarticular tuberculosis was 100% confirmed on the basis of histopathology.

## Discussion

Our study was prompted by the observation that meta-diaphyseal osseous tuberculosis of long bones is often difficult to distinguish from bone tumors based on clinico-radiological features due to the non-characteristic presentation. In a diagnostic dilemma, the consequences of misdiagnosing a malignancy as non-malignant process are graver than a missed diagnosis of infection. Therefore, these patients tend to be referred to tumor centers to solve the diagnostic challenge.

The ability of the orthopedic oncologist to correctly predict tuberculous infection based upon clinical examination and radiological features was 60%, while consensus review of imaging with a musculoskeletal radiologist improved the predictive ability to 72% comparing the radiological and clinical features of the referred patient.

Tissue diagnosis remains the mainstay for definitive diagnosis in a diagnostic dilemma.

**Figure 1:**
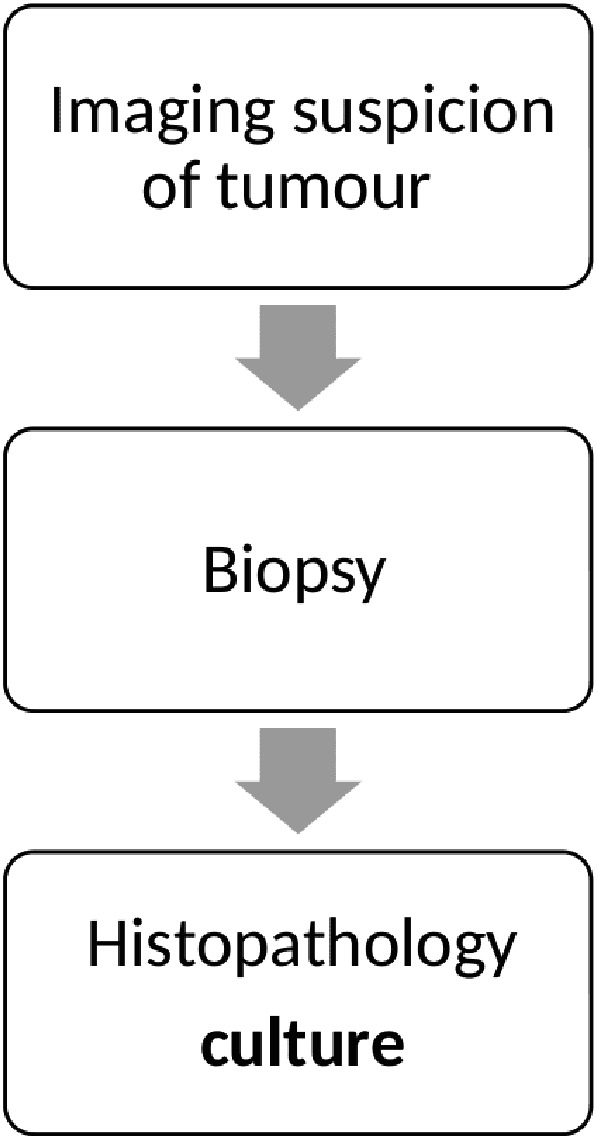
Simple flowchart of the diagnostic protocol to confirm tuberculosis.

**Figure 2:**
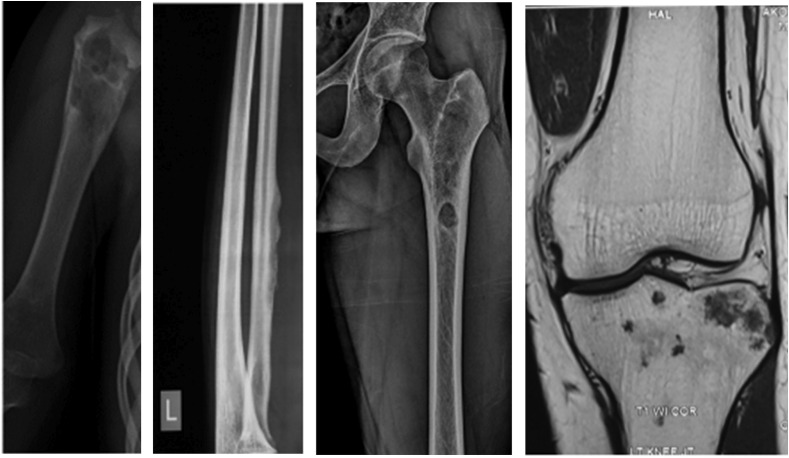
Patients referred based only on imaging diagnosed as unicameral bone cyst, periosteal osteogenic sarcoma, osteoid osteoma, and metastasis; the diagnosis was, however, confirmed on histopathology.

**Figure 3:**
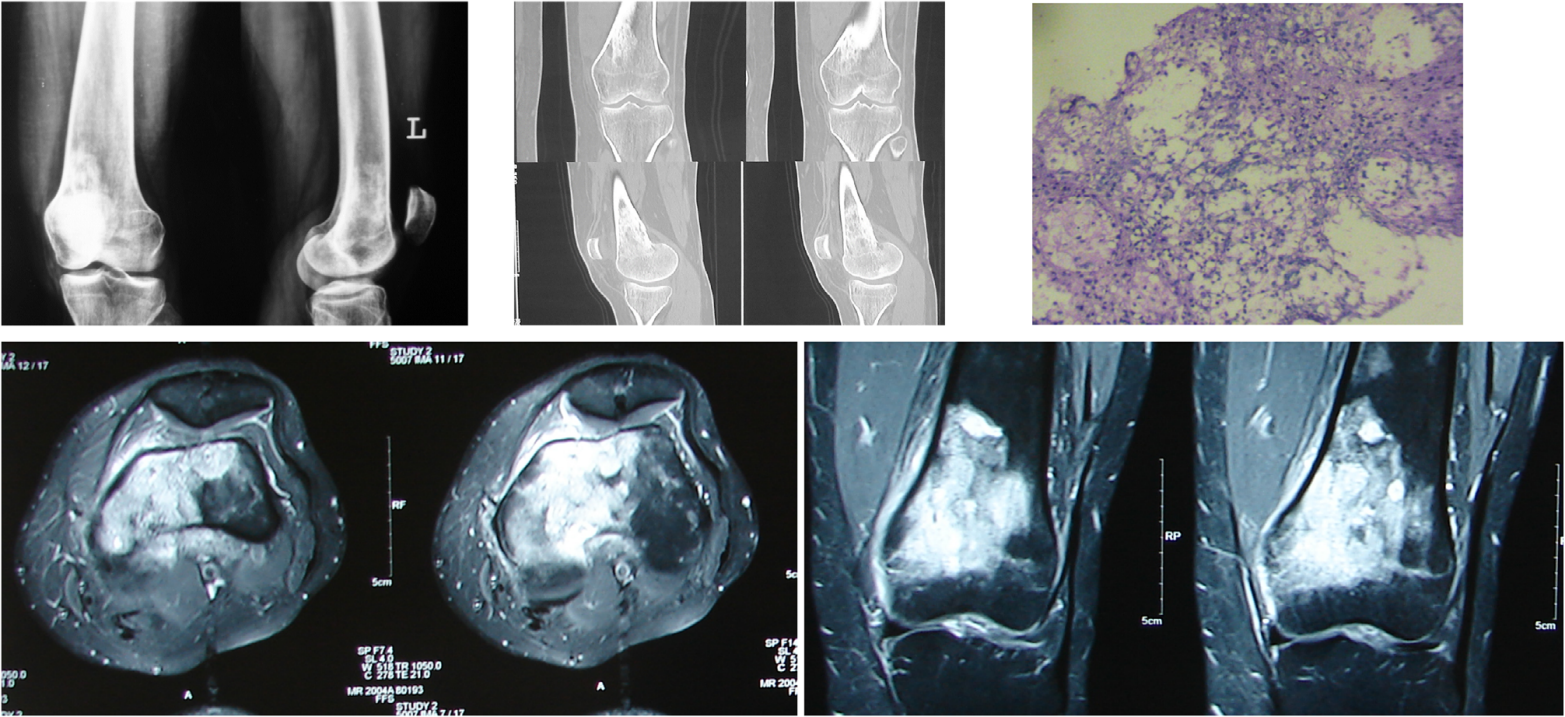
A 24-year-old female referred to our center with a history of mass progressively increasing in size over the past 3 months with a history of weight loss and progressive pain. X-ray shows a dense sclerotic lesion with enhancing marrow oedema on the MRI. The final diagnosis was based upon histopathology.

**Figure 4:**
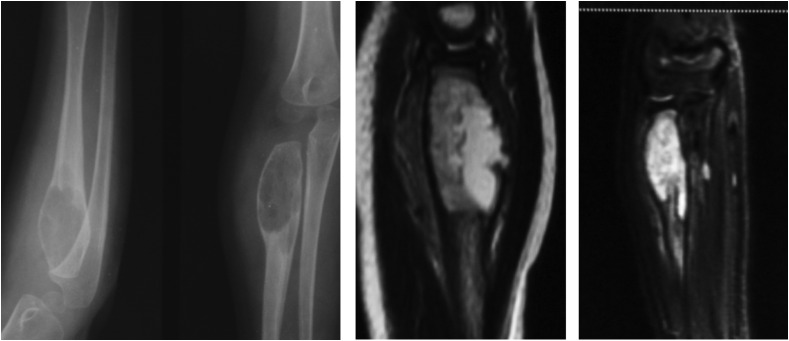
A 6-year-old female with a mass over the elbow and restricted range of motion was referred with an X-ray suggestive of a bone cyst. An MRI of the swelling showed a non-specific enlargement with a break in the cortex, a biopsy and histopathology confirmed the presence of tuberculous infection in this patient.

As a protocol followed at our institute, all biopsies were sampled for culture as well as histopathological analysis as mentioned in the flowchart below (Figure 1). All biopsies were obtained by a core needle and under image guidance (Figure 2) to target the lesion. A frozen section analysis was added at time of needle biopsies to confirm representative pathological tissue; thereby eliminating a need for repeat biopsies in all except 2 cases (Figures 3 and 4) which underwent an open biopsy prior to referral.

Final diagnosis of tuberculous osteomyelitis is made on the basis of histopathological tissue diagnosis, tissue cultures demonstrating *Mycobacterium tuberculosis* growth as the gold standard [6, 7]. Histopathological diagnosis demonstrating AFB on ZN Stain and presence of epithelioid granulomas with caseous necrosis aid the diagnosis in the absence of positive cultures.

Osteo-articular tuberculosis being paucibacillary, the yield of cultures tends to be low. It is therefore essential all tissues collected on biopsy be tested for culture as well as histopathology; as is the protocol followed at our institute.

The radiological signs in long bones (mainly diaphyseal) can vary according to age of the patient, duration of the disease, and region of involvement. There are no predefined radiological features of T.B. Osteomyelitis of long bones [6]. The musculoskeletal radiologist at our center has listed the clinical features under Table 1.

**Table 1:** Percentage of each radiological feature observed by the musculoskeletal radiologist and orthopedic oncologist.

Radiological features	Percentage
Joint involvement	68
Metaphyseal involvement	40
Wide Transition zone	68
Codmans triangle	4
Periosteal reaction	36
Soft tissue mass	60
Cortical involvement	56
Permeative lesion	40

On radiographs the essential features pointing toward a tumor would be a Codman’s triangle and a breach in the cortex along with a large soft tissue mass, whereas Tuberculosis presents with a metaphyseal involvement and a permeative cortical lesion with a relatively small soft tissue shadow which may indicate a soft tissue involvement or a surrounding cold abscess.

An early modality for the diagnosis of tuberculous osteomyelitis is the MRI. It demonstrates intraosseous involvement earlier than the other imaging modalities. Marrow changes are demonstrated as areas of low and high signal intensity on T1W- and T2W-weighted images, respectively, and show enhancement after intravenous administration of gadolinium chelates. Areas of necrosis appear hypointense on T2W images and do not show enhancement [8, 9]. Majority of the tumors, on contrast MRI show a uniform uptake of dye; however, it has been observed that in case of tuberculosis only the peripheral rim of cells shows an enhancement in our study.

Gallium-67 citrate, Indium-111–labeled autologous leukocyte (white blood cell) scintigraphy, and fluorodeoxyglucose (FDG) positron emission tomography (PET) are very useful in the diagnosis of extrapulmonary tuberculosis [13, 14]. Studies have shown that it is more sensitive than other modalities for diagnosis of infection in its early stages [7]. However, this modality of investigation in diagnosis of extrapulmonary tuberculosis is still in its infancy and needs further study to determine its efficacy. The author has no personal experience in using the above modalities for diagnosis.

Recent studies suggest that use of superparamagnetic iron oxide (SPIO) MRI contrast agent for bone marrow imaging appears promising, for differentiation between inflammatory lesions and neoplastic lesions compared with the standard gadolinium complex MRI scans [8].

We also analyzed laboratory reports of the following: the erythrocyte sedimentation rates (ESR), C-Reactive Protein (CRP), serum alkaline phosphatase, and white blood cell (WBC) count.

The CRP values were negative in 60% of the patients.

The average ESR value in our study was 26.6 mm/hr. which is slightly above the normal range, the WBC count was within the normal range for all the patients with an average of 7377.7 (Normal range 4000–10,000 WBCS/mm^3^).

**Figure 5:**
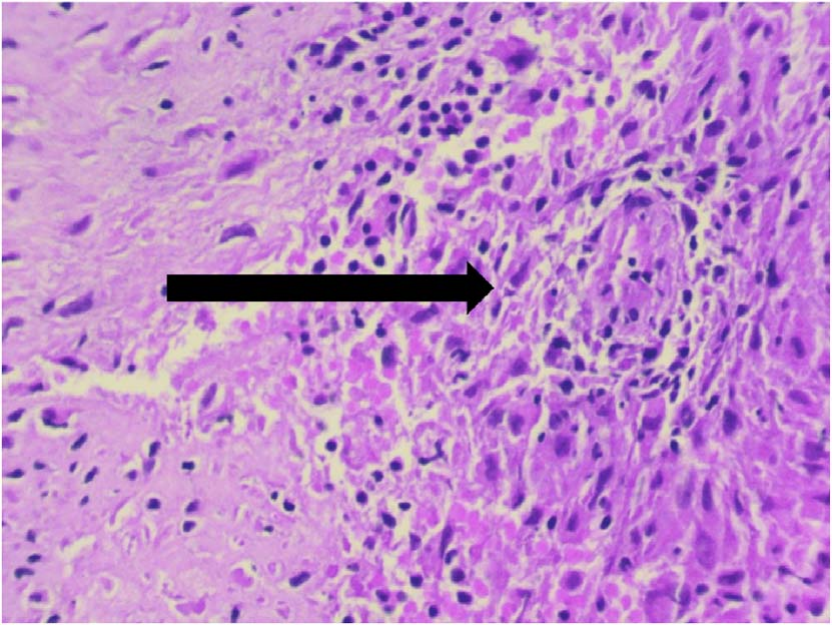
Langhans giant cells demonstrated by an arrow.

The final diagnosis, however, is made on the basis of the histopathology if cultures fail to give a positive result. The occurrence of tuberculosis in long bones, even though very rare, gives a classical picture of epithelioid granuloma with caseous necrosis. The presence of Langhans giant cells makes the diagnosis of tuberculosis even more likely as shown in Figure 5. Two patients needed a repeat biopsy (both biopsies performed at other institutes) as a result of poor fixation, non-representative tissue, and wrong staining techniques. It is hence imperative to have a good histopathology set up with an experienced pathologist.

It is important that a center doing high volumes of biopsies must have a pathologist trained in frozen sections. A representative tissue sample obtained from a frozen section must be confirmed before it is sent for final histopathological study [6]. Materials obtained from non-representative areas may lead to errors in final diagnosis apart from increasing the cost as well as the donor site morbidity.

Osteoarticular tuberculosis is a paucibacillary disease hence the demonstration of acid fast bacilli in samples obtained from biopsy is very low compared to other diagnostic modalities like PCR [9]. The average time to detect the presence of tuberculous bacilli is 6.5 h in case of PCR compared to cultures that may take approximately 3 weeks.[10]. Although the gold standard for the diagnosis of tuberculosis is demonstration of acid fast bacilli, it has poor sensitivity compared to PCR [11]. Mycobacterium tuberculosis remains the main causative organism and only a few cases are attributable to *Mycobacterium Bovis*. Atypical mycobacteria, such as *Mycobacterium kansasii*, *Mycobacterium marinum*, *Mycobacterium scrofulaceum*, and *Mycobacterium avium* complex account for approximately 1–4% of cases of tuberculosis. Tuberculin skin test (TST) and IFN-γ releasing assay (IGRA) may be the supportive method for diagnosing tuberculosis, but it has a limited diagnostic value, especially in regions where the disease is endemic or with previous exposure to BCG vaccination.

Diagnosis based primarily on the basis of imaging is a wrong approach to any aggressive looking lesion. However, over the past 8 years, we have had numerous cases referred to our institute on the suspicion of being a tumor based primarily upon radiology

Only two patients had a biopsy done for the lesion before they presented to our institute. Both biopsies were inconclusive and required a second biopsy for confirmation.

The biopsy should yield a material that is sufficient for histopathology and microbiological studies. The biopsy should preferably be done by the final operating surgeon [12]. Biopsy must always be along the final surgical incision, a wrongly done biopsy may have a catastrophic outcome on the final surgery as unplanned biopsy may lead to spillage and contamination of the biopsy tract.

## Conclusion

It is essential to have a multidisciplinary approach to any lesion of the bone, particularly if it mimics a sarcoma. Even a benign looking lesion should be assumed to be malignant and a complete work up as shown in the flowchart should be adopted.

Lesions with a wide zone of transition, absent Codman’s triangle with sclerotic rims with minimal periosteal reaction, should raise the suspicion of T.B. unless proven otherwise. It also essential to have a team of pathologists trained in sarcoma diagnosis to be able to give a definitive diagnosis. An infection control team is an essential part of patient management. It is important to send every histopathology specimen for culture and every culture specimen for histopathological analysis for comprehensive management of the patient.
